# Perceptions and experiences of skilled birth attendants on using a newly developed strap-on electronic fetal heart rate monitor in Tanzania

**DOI:** 10.1186/s12884-019-2286-7

**Published:** 2019-05-10

**Authors:** Sara Rivenes Lafontan, Hussein L. Kidanto, Hege L. Ersdal, Columba K. Mbekenga, Johanne Sundby

**Affiliations:** 1 Institute of Health and Society, Faculty of Medicine, University of Oslo, Forskningsveien 3A, 0373 Oslo, Norway; 2 Medical College, East Africa, Aga Khan University, Dar es Salaam, Tanzania; 3 Department of Research, Stavanger University Hospital, Postboks 8100, 4068 Stavanger, Norway; 4 Department of Anesthesiology and Intensive Care, Stavanger University Hospital, Postboks 8100, 4068 Stavanger, Norway; 5 Faculty of Health Sciences, University of Stavanger, 4036 Stavanger, Norway; 6 School of Nursing and Midwifery, Aga Khan University, Dar es Salaam, Tanzania

**Keywords:** Health care providers, Fetoscope, Doppler, Fetal heart rate, Labor, Moyo, Tanzania, Low-resource setting, Newborn health, Midwives

## Abstract

**Background:**

Regular fetal heart rate monitoring during labor can drastically reduce fresh stillbirths and neonatal mortality through early detection and management of fetal distress. Fetal monitoring in low-resource settings is often inadequate. An electronic strap-on fetal heart rate monitor called Moyo was introduced in Tanzania to improve intrapartum fetal heart rate monitoring. There is limited knowledge about how skilled birth attendants in low-resource settings perceive using new technology in routine labor care. This study aimed to explore the attitude and perceptions of skilled birth attendants using Moyo in Dar es Salaam, Tanzania.

**Methods:**

A qualitative design was used to collect data. Five focus group discussions and 10 semi-structured in-depth interviews were carried out. In total, 28 medical doctors and nurse/midwives participated in the study. The data was analyzed using qualitative content analysis.

**Results:**

The participants in the study perceived that the device was a useful tool that made it possible to monitor several laboring women at the same time and to react faster to fetal distress alerts. It was also perceived to improve the care provided to the laboring women. Prior to the introduction of Moyo, the participants described feeling overwhelmed by the high workload, an inability to adequately monitor each laboring woman, and a fear of being blamed for negative fetal outcomes. Challenges related to use of the device included a lack of adherence to routines for use, a lack of clarity about which laboring women should be monitored continuously with the device, and misidentification of maternal heart rate as fetal heart rate.

**Conclusion:**

The electronic strap-on fetal heart rate monitor, Moyo, was considered to make labor monitoring easier and to reduce stress. The study findings highlight the importance of ensuring that the device’s functions, its limitations and its procedures for use are well understood by users.

**Electronic supplementary material:**

The online version of this article (10.1186/s12884-019-2286-7) contains supplementary material, which is available to authorized users.

## Background

Globally, an estimated 2.6 million macerated and fresh stillbirths (FSB), and 2.7 million newborn deaths (28 days) occur each year [1, 2]. Approximately 40% of stillbirths and early newborn deaths occur in relation to birth, with 1.3 million FSB and 1 million newborn deaths annually [1, 2]. Most of these deaths share a common hypoxic-ischemic pathway (birth asphyxia) and are preventable by early recognition of fetal heart rate abnormalities coupled with timely obstetric interventions and deliveries [3]. Fetal heart rate monitoring (FHRM) is considered a key component of intrapartum care in order to detect signs of fetal distress and a potentially hypoxic fetus [4, 5]. However, studies from Tanzania revealed that FHRM is not conducted as frequently as recommended, which may cause unnecessary perinatal morbidity and mortality [6–8]. FHRM can be carried out intermittently or continuously.

Intermittent auscultation (IA) is the recommended method of FHRM for women with a low risk of complications during labor. In low-resource settings IA is commonly carried out using a Pinard fetoscope. However, Pinard is reported to be difficult to use, time-consuming, and painful for the mother [9, 10]. An alternative electronic solution is the hand-held Doppler ultrasound that is believed to cause less pain, be easier to handle and more reliable [11, 12]. According to international guidelines IA should be performed every 15–30 min during first stage of labor and every 5–15 min during the second stage [13, 14]. This requires a ratio of skilled birth attendant to laboring woman of 1:1 or 1:2, which is rarely the case in most low-resource settings, characterized by a lack of skilled birth attendants to adequately monitor each fetus [15]. While there remains a debate about the efficacy of continuous FHRM by cardiotocography (CTG) to prevent adverse perinatal outcome, it is recommended for use in high-risk pregnancies and is commonly performed in high resource settings [16]. Due to a variety of challenges related to price, access to electricity, maintenance and training of staff in the interpretation of CTG traces, it is rarely used in labor wards in low-resource settings.

**Fig. 1:**
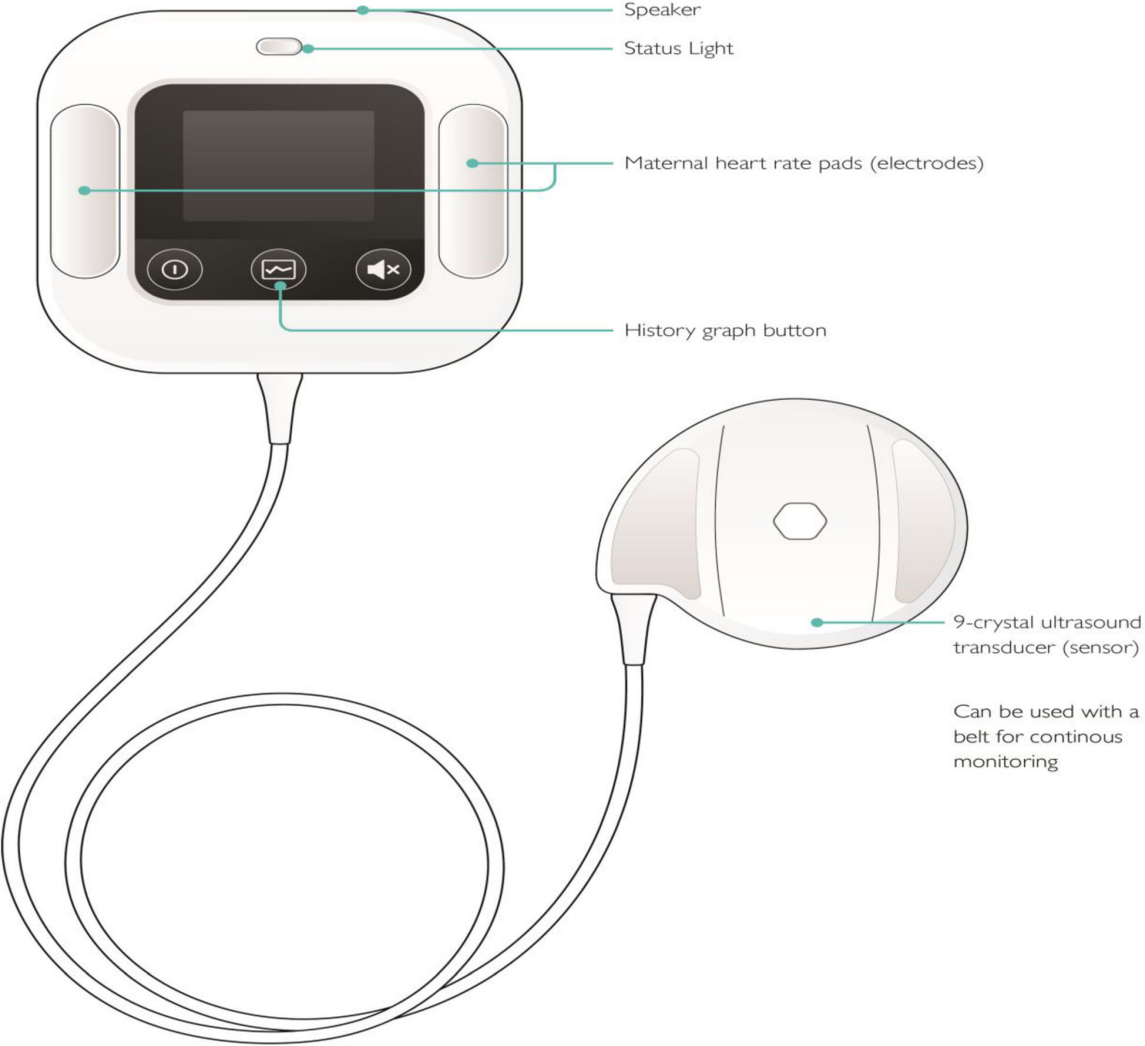
The electronic fetal heart rate monitor, Moyo and its characteristics. Credit: Laerdal Global Health, no copyright

In an effort to improve FHRM in low-resource settings and to enable more timely responses to detection of an hypoxic fetus, Laerdal Global Health developed a new automatic FHR monitor with a nine-crystal Doppler ultrasound sensor, called Moyo (Fig. 1). It can be strapped to the women’s abdomen and detect FHR intermittently or continuously. It alerts the birth attendant when detecting an abnormal FHR and stores Doppler signal for subsequent analysis. Moyo was introduced in Tanzania in 2015 and was associated with improved FHRM and abnormal FHR detection in quantitative studies [12, 17]. There is limited knowledge about the views of health care providers using electronic FHRM devices in low-resource settings. One qualitative study conducted among midwives in Tanzania found that while electronic FHRM was considered to have many advantages, challenges were related to insufficient training and follow-up concerning the correct use of the device, and a lack of trust in its reliability compared to Pinard [18]. In another qualitative study from Tanzania, skilled birth attendants perceived both the training received prior to using Moyo and in particular training concerning the correct follow-up actions to the device’s abnormal FHR alerts to be inadequate [19]. Increased knowledge about the experiences of skilled birth attendants about the use of new technology in labor care is important in order to ensure successful adoption in low-resource settings which in turn may lead to a reduction in morbidity and mortality. The objective of the present study was to explore the attitude and perceptions of birth attendants using Moyo. This study is the qualitative component of an evaluation of the introduction of Moyo in two urban hospitals in Dar es Salaam, Tanzania [12].

## Methods

### Study design

The study employed a qualitative design drawing on semi-structured in-depth interviews (IDIs) and focus-group discussions (FGDs) with skilled birth attendants to explore attitudes toward labor monitoring and experiences using different methods of fetal heart rate monitoring.

### Setting

The study was performed at two tertiary health care facilities in Dar es Salaam. This is the largest city in Tanzania and has a population of approximately 3,4 million. The maternity ward at Hospital 1 receives both public and private patients. The hospital receives referral patients from the entire city of Dar es Salaam and neighboring regions. About 10,000 deliveries take place annually, of which 50% are caesarean sections (CS) according to one study [20], the highest rate in the country. The maternal mortality rate at the hospital averaged 301/100,000 live births from 2013 to 2015 and the stillbirth rate was 78/1000 live births at the facility [21]. The obstetric department is staffed with a number of obstetric and gynecologic specialists, resident doctors, intern doctors and nurse/midwives. The labor ward includes 19 beds and five nurse/midwives per shift in addition to specialists in obstetrics, resident doctors and intern doctors. Hospital 2 receives obstetric referrals from facilities within its catchment area, which covers one of three districts of Dar es Salaam. About 17,000 deliveries are performed annually, with a CS rate of 6.5% [22]. Two specialists in obstetrics work in the maternity ward in addition to medical doctors, intern doctors and nurse/midwives. The labor ward has 12 beds and five nurse/midwives working during the day.

### Participants and data collection

The data collection was conducted between January and March 2017. Invitations to participate in the study were issued to both nurse/midwives and medical doctors working in the maternity ward at the two hospitals and who had used Moyo. Efforts were made to include participants who varied in age, gender and number of years they had worked at the labor ward in order to capture a broad spectrum of perspectives on the study objective. In total, five FGDs were conducted and 10 semi-structured interviews were carried out. All interviews and FGDs were conducted in Kiswahili by a research assistant who is also a midwifery teacher, in the presence of the first author. Three semi-structured interviews were conducted in English by the first author. The five FGDs were conducted prior to the interviews and were arranged by cadre, with one FGD composed of all the medical doctors who participated in the study. Participants from the FGDs were then selected for an additional semi-structured in-depth interview. An interview guide was used for each FGD and IDI (Additional files 1 and 2 respectively). This included the objective, and open-ended questions regarding fetal heart rate monitoring, collaboration between staff at the labor ward and instances when Moyo was used. When it was believed that thematic saturation was reached and no new themes arose, an additional FGD and IDI was carried out in order to validate findings with new respondents- a process often referred to as respondent validation or member checking [23]. These IDIs and FGDs were part of the total number carried out. Each interview or FGD took place at the health facility in an area where privacy could be ensured. Each FGD lasted approximately 60–90 min; the IDIs approximately 40–60 min. All were audio-recorded.

### Data analysis

Data were transcribed on an on-going basis by a transcriber with experience in transcribing qualitative data and who had also been trained by the first author. The transcripts were then translated into English by a bi-lingual Kiswahili/English speaker. Translations were discussed with the first author for clarity. The first author transcribed the interviews conducted in English. Field notes were taken during the entire data collection and included in subsequent analysis. A qualitative content analysis was used to analyze the data. The analytical process included assigning descriptive codes to the transcribed material using NVivo 11 Software by the first author. The codes were subsequently merged into categories as described by Graneheim and Lundman [24, 25]. The transcribed material, including codes and categories was discussed and agreed among authors. Table 1 illustrates how the transcribed text was condensed into codes and categories.

**Table 1:** Example of the analytical process

Transcribed text	Code	Category
*When you decide there is a fetal distress and you decide to do a C-section, the time for preparation is too long. You might say this is fetal distress then pass half an hour, forty- five minutes and still they are in the labor ward.*	Delays	Feeling overwhelmed and unable to provide optimal labor care prior to using Moyo

### Intervention

Moyo was introduced at the two study sites in Dar es Salaam in 2015. Each study site received approximately 35 devices each. Training sessions concerning the functions of the device and indications for use were organized for staff. Details of the training have been described elsewhere [19]. Randomized control trials comparing the device to other methods of FHRM were also conducted at each study site. After the completion of the trials, the devices are currently used in the labor wards.

## Results

### Participants’ characteristics

In total, 28 nurse/midwives and doctors participated in the study: 24 females and four males. The mean age was 37 and the median time of working in the labor ward was 4.8 years, with a range of 1–12 years.See Table 2 for overview of data collection and participants profession by study site.

**Table 2:** Overview of data collection and participants profession by study site

	Data Collection	Medical Doctor	Nurse/midwife^a^
Study site 1	3 FGDs 5 IDIs	0	12
Study site 2	2 FGDs 5 IDIs	7	14

^a^Five of the nurse/midwives from each study site participated in semi-structured in-depth interviews (IDIs) in addition to the focus group discussions (FGDs)

The attitudes and perceptions of skilled birth attendants towards using Moyo were organized in four categories: 1) Feeling overwhelmed and unable to provide optimal labor care prior to using Moyo, 2) Moyo facilitated ease and reduced stress of monitoring; 3) Perceived insufficient training leading to challenges implementing Moyo; and 4) the perceived benefits for the laboring women of using Moyo.

### Feeling overwhelmed and unable to provide optimal labor care prior to using Moyo

The context within which Moyo was introduced was described as both challenging and characterized by a high workload, which left the nurse/midwives feeling overwhelmed on a regular basis. In particular, the number of laboring women who required monitoring and the lack of resources to provide good care were frequently mentioned. It was also mentioned that it was difficult to conduct FHRM as frequently as recommended:

Participant: *You become lazy when using Pinard, having to listen to the fetal heartbeat every half an hour. I must tell the truth, we never used to do that often.*

Interviewer: *What was the problem?*

Participant: *Not enough time, had a lot of patients to see.*

(Nurse/Midwife 3, IDI, Hospital 2)

The difficulties of documenting the FHRM of each patient in the partogram before the introduction of Moyo was also mentioned as an issue:

*Sometimes we only examine…and only record if the contractions are severe. Recording every half hour is not realistic.*

(Nurse/Midwife 4, FGD 1, Hospital 2)

During instances of fetal distress, the collaboration between the midwives was highlighted as crucial both to assist in the situation and to avoid blame for a potentially negative outcome. This was not a topic included in the interview guide but mentioned by both doctors and nurse/midwives:

*If there is a fresh stillbirth or early neonatal death, you’re in trouble. We tell each other “whenever you face a difficulty when conducting deliveries, if you see a patient is changing condition, just shout.” Somebody will call someone else. Three people will be there. You help each other so if something bad happens, all of you guys are there. So three heads can’t miss out things. So if something bad happens, it was unavoidable.*

(Nurse/Midwife 3, IDI, Hospital 1)

Coping with the emotional effects of poor fetal outcomes were also an issue raised by the participants. One participant described being heartbroken after the delivery of a FSB:

*The baby was very fragile when she came out. I felt bad because the fetal heart rate wasn’t normal from the beginning to the end and then on top of all that all those other problems developed. It broke my heart.*

(Nurse/Midwife 5, IDI, Hospital 2)

### Moyo facilitated ease and reduced stress of monitoring

Some of the participants described a process of going from feeling uncertain about Moyo and its benefits when it was first introduced, towards a gradual appreciation of its benefits after becoming more comfortable with the device.

*Now I trust Moyo very much, although in the beginning I did not know how to operate it properly. Now after being conversant with it, I trust it so much and it is so accurate.*

(Nurse/Midwife 2, IDI, Hospital 2)

One participant felt she was missing the connection with the fetus that was provided by the Pinard fetoscope when using Moyo, a connection she described as being developed by staying and listening to the FHR:

*You feel a connection to the baby because once you listen, it’s like magical. You stay and listen and after some time the baby is out. There is some sort of connection. With Moyo you just put it on and you hear. So you don’t feel like you are really connected.*

(Nurse/midwife 3, IDI, Hospital 1)

Overall, Moyo was considered an advantage to the majority of participants, both doctors and nurse/midwifes. One of the positive aspects most commonly mentioned was that Moyo made their work easier by making it possible to monitor several women at the same time. The knowledge that they would be alerted in cases of fetal distress was mentioned by several of them as reducing the stress of monitoring a number of laboring women at the same time:

*The alarm function makes you relaxed. Once the alarm goes off you go and check. If anything causes stress it is Pinard because it takes time to locate the pulse and the results are not always accurate.*

(Nurse/midwife 5, FGD 3, Hospital 1)

Participants reported reacting faster to alerts of abnormal FHR alerts when using Moyo compared to Pinard. There were two reasons for this; The first was the fact that all staff were alerted to instances of fetal distress at the same time, and would rush to the mother because of the alarm; the second reason was that as everyone was able to see the FHR on the device’s display, and considered it to be both more reliable and less subjective than Pinard where only one person at a time can hear the FHR. This latter point was often mentioned by the doctors who experienced more difficulties hearing the FHR when using Pinard:

*When I use Pinard my actions are slower than when I use Moyo because Moyo gives me the exact reading. When I use Pinard I may hear and say maybe I did not hear well and I will call a colleague to come and hear. So delays in making a decision. Because I will call the midwife to come and listen. But with Moyo, you make a decision straight away.*

(Doctor 2, FGD, Hospital 2)

Overall, it was felt that Moyo had contributed to improved fetal outcomes at the labor wards. The device was said to be particularly useful in instances of complicated cases and when the FHR was not found using Pinard. Several participants told stories of how they believed the fetus had survived because Moyo was used:

*I attached Moyo which indicated that there was fetal distress. I told the patient that her fetal heart rate was abnormal and that we would do our best to save the baby, so I gave her Ringer lactate but the fetal heart rate was still abnormal after half an hour so I called the doctor. He told me to look for a doctor who would perform an operation but I wanted to try once more, so I continued to monitor the fetal heart rate and gave another dose of Ringer lactate and within one hour the fetal heart rate went back to normal. When I examined her she was in second stage of labor so I encouraged her to push. She delivered the baby, though the baby was born with the cord around the neck but I said to myself that without Moyo the baby would not have survived.*

(Nurse/Midwife 4, IDI, Hospital 2)

### Perceived insufficient training leading to challenges implementing Moyo

There was a lack of clarity concerning when and how Moyo should be used. The nurse/midwives gave different answers to questions about indications for continuous FHRM, with some believing that all mothers should wear the device for continuous monitoring.

Another issue frequently mentioned issue was related to the function of the device in measuring the maternal heart rate. The participants admitted that it was rarely used, even in instances when the device indicated an abnormally low FHR. During one FGD with six participants, one participant said she believed it was not used often enough, and then went on to ask each of the other participants how many times they had used it. Of the six, one admitted never having used the function and two reported using it only once.

Both nurse/midwives and doctors had experienced seeing the FHR on the display but later delivering a stillbirth. One participant raised it as a possible explanation as to why some colleagues were reluctant to use Moyo. Variations of the following account were told by several participants both in FGDs and during IDIs at both study sites:

*I got a fetal heart rate from Moyo and convinced myself that the fetal heart rate was there. The woman was having a ruptured placenta so I rushed her for CS but on delivery the fetus was not alive.*

(Doctor 5, FGD, Hospital 2)

One issue that was mentioned in relation to this account, was the lack of monitoring when the mother left the labor ward for surgery, and the waiting time from between referral until the procedure was carried out. One midwife explained how Moyo was removed before leaving the ward to go to the operating theatre for CS:

*When the mother is having a Cesarean and on the way to the theatre for operation, we normally remove Moyo, patients don’t take them to the theatre.*

(Nurse/Midwife 4, FGD 2, hospital 1)

While stating that the device was easy to use, the participants also believed there should have been more training concerning how to use Moyo. It was often mentioned that the device was not turned off between patients and that procedures for cleaning and charging the device were not followed resulting in insufficient availability of devices at any given time.

### The perceived benefits for the laboring women of using Moyo

Moyo was believed to contribute positively to the provision of care. Some participants mentioned that it increased communication between the midwife and the laboring woman.

Moyo was also perceived to be more comfortable for the woman compared to Pinard. Despite some reports of complaints from mothers that it was uncomfortable to wear the belt, the participants seemed to perceive Moyo as being something positive for the women:

*We tell the mothers that we use Moyo because it is more efficient. They are generally happy to be able to see the progress of their baby on the screen.*

(Nurse/Midwife 2, IDI, Hospital 1)

One participant also believed it attracted more women to deliver at the hospital:

*Moyo is a new device, we only saw it for the first time when we were trained. Now the word is out on the street that there is a new device at ... [ Hospital 2] and therefore people come to this hospital because of this new device.*

(Nurse/midwife 4, FGD 2, hospital 2)

## Discussion

The present study explored the perceptions and experiences of skilled birth attendants using an electronic FHRM monitor called Moyo. The participants in the study considered Moyo a useful tool to improve FHRM. Issues related to the use of Moyo included a lack of clarity about the correct use of the device. The context in which Moyo was implemented also seemed to affect adoption of the device.

Prior to the introduction of Moyo, participants in the study described being unable to provide adequate labor monitoring and documentation due to a lack of resources. They also described a strong sense of collaboration and support between each other in order to avoid being blamed for poor fetal outcomes and the associated consequences. We hypothesize that the combination of a lack of resources to provide adequate labor care and a fear of being blamed for-and coping with-negative fetal outcome might cause high levels of stress, which in turn affect patient care. A systematic review among health care providers in low-and middle-income countries found burnout to be highly prevalent [26]. However, the participants in the study described both feeling more relaxed when Moyo was used knowing that the alarm would alert the birth attendants of fetal distress, and an overall feeling that the device made the work easier. This aspect of electronic continuous FHRM was also mentioned in a mixed- methods study from Uganda about perceptions of a prototype CTG used on non-laboring pregnant women at term [27]. Thus, the use of what the participants in the present study perceived to be a crucial tool in performing their duties seemed to reduce stress, which could lead to increased job satisfaction; similar to findings from a study investigating the effects of new technology among health care providers in South Africa [28].

Both nurse/midwives and doctors experienced fear of the consequences of being blamed for negative maternal or fetal outcome, in line with findings from a qualitative study from Tanzania [29]. In the present study the nurse-midwives described ways in which they would try to avoid this blame by always working as a team of midwives if the condition of the laboring woman or fetus worsened. Quantitative studies from Tanzania comparing the continuous use of Moyo to intermittent use of Pinard fetoscope, did not find an improvement in perinatal outcome despite an increased identification of abnormal FHR, in part due to delays in obstetric follow-up actions [12, 30]. Additionally, a qualitative study among birth attendants in Tanzania about the training they received prior to using Moyo found uncertainties among the nurse/midwives concerning how to respond to alerts of fetal distress [19]. The fear of being blamed for negative outcomes, stress and uncertainties about how to respond to fetal distress alerts combined with systemic context barriers, could explain why increased detection of fetal distress through electronic FHRM have not so far been shown to result in reduced mortality rates [31].

Several participants, both nurse/midwives and doctors, told similar accounts of how Moyo had indicated a FHR but the fetus was delivered as a FSB. These accounts illustrates some of the challenges using Moyo raised, such as lack of a clarity about procedures for use and insufficient training. The fear of being held responsible for poor outcomes could explain instances of blame avoidance and dislocating responsibility to Moyo not working properly. While a culture of “naming and blaming” [32] is difficult to change, it might have been beneficial to analyze and discuss these events in an early phase of Moyo’s introduction. Using the account as a topic of discussion could also have increased awareness about the limitations of the device and reinforced procedures for use.

The account of the fresh stillbirth was mentioned by some participants in relation to the issues of occurring after referral to surgery. At both hospitals, Moyo was removed when the patient left the labor ward for cesarean-section (CS) and delays after CS referral were reported as common, with limited FHRM during the waiting time. This could lead to a poor outcome of severely asphyxiated fetuses. At the time of data collection, Hospital 1 had a surgical theatre used exclusively for obstetric emergencies, whereas Hospital 2 did not; there, obstetric emergencies were dealt with in the general surgical theatre and were dependent on capacity. Studies from Tanzania indicate that unnecessary CS is already a challenge in tertiary health facilities in the country [21, 29, 33]. As quantitative studies found an increase in CS after the introduction of Moyo [17], addressing the factors contributing to the overuse of CS is becoming paramount.

The participants felt using Moyo made their work easier, allowing for several women to be monitored at the same time and knowing that they would be alerted in case the device detected an abnormal FHR. While continuous FHRM is not recommended for low-risk pregnancies due to adverse effects such as the increase in CS, calls have been made for innovative alternatives to CTG such as Moyo to improve “intermittent prolonged” FHRM in low-resource settings, in part due to a “substantial mismatch.. between international guidelines and what is locally achievable” [15]. Indeed, it is difficult to imagine how the five skilled birth attendants working per shift at Hospital 2, are able to perform IA every 15–30 min on each of the 46 women delivering at the hospital every day. This reality highlights the urgent need to increase the number of skilled birth attendants and address other systemic barriers [34, 35]. It also illustrates the need for locally adapted, context-specific solutions developed in close collaboration with users. In Zanzibar, locally adapted intrapartum guidelines which were developed and implemented in a participatory process with skilled birth attendants resulted in significant improvements in newborn survival [36, 37].

Several participants reported how the maternal heart rate was not always measured during detection of abnormal FHR. Intrapartum misidentification of maternal heart rate as FHR is well known, particularly when the maternal HR is above 100 beats/minute [38, 39]. The International Federation of Obstetrics and Gynaecology (FIGO) recommends simultaneous evaluation of the maternal pulse when using handheld Doppler and reviewing the sound from the device [39]. Furthermore, when using any Doppler device there is a slight risk that the device may sporadically indicate a number that could be interpreted as FHR due to movement by the mother or abdominal movements such as fluids or muscles. FIGO guidelines recommend that users listen for the rhythmic sound mimicking the fetal heart, described in the guidelines as a *galloping horse* to avoid misidentification. Misidentification could be another possible explanation for the account of the FSB and further illustrates the importance of using the story as a topic for discussing the device’s procedures for use. We recommend that steps to avoid misidentification of FHR should be emphasized in training birth attendants about the use of Doppler devices. Due to the lack of knowledge of this issue in similar settings, we believe it could be an area for further investigation.

The process of becoming conversant in using Moyo was described by some as one of initial skepticism towards the device with a growing trust in it after becoming confident in its use.

Some of the participants believed Moyo was more accurate and less subjective than Pinard; which was expressed as an almost blind trust in the device and its ability to detect fetal distress. This might be one of the reasons why procedures such as verification the FHR with Pinard and using the maternal HR function were reported as often being ignored. A study from Tanzania investigating perceptions among birth attendants about ultrasound use, found an overestimation of the benefits of ultrasound during labor and a possible reduction in attention to other methods of FHRM [40]. Thus, there seems to be a risk that the adoption of a new device may result in the reduced use of current tools, such as the Pinard fetoscope. Conversely, some participants described missing the connection they gained with the fetus when using the familiar Pinard which is similar to a finding from a study investigating perceptions about a FreePlay wind-up Doppler among midwives north in Tanzania [18]. This indicates the complexity of the adoption of new technology in labor care that we believe should be investigated further.

### Limitations

The findings of the study need to be considered in relation to its limitations. Those who agreed to participate in the study were overall positive towards the device and thus might have been more motivated to participate in the study than those with negative experiences who did not want to use Moyo. While participants were informed before consenting to agree to take part in the study that raising negative perceptions or experiences of using Moyo would have no repercussions - an assurance that was also repeated during the data collection - there is a risk that participants may have expressed more positive views for fear of repercussion if they did not. An attempt to mitigate this issue was to interview some of the participants twice by inviting them to take part in a semi-structured interview after the FGD. This was carried out in order to build trust with the participants, to allow for further reflection on the issues discussed and to improve the quality of the data [41]. Further investigations are needed into the perspectives of skilled birth attendants who reject devices such as Moyo. The participants worked at two tertiary facilities with more exposure to new technology than skilled birth attendants working in smaller primary health care facilities who might be more hesitant towards using Moyo.

## Conclusion

The electronic strap-on FHRM Moyo was perceived to make labor monitoring easier and to reduce stress among birth attendants, who, prior to the introduction of the device, described feeling overwhelmed by a high workload and an inability to adequately monitor each laboring woman. Challenges associated with its use included a lack of clarity concerning procedures and indications for use. The study findings highlight the importance of ensuring that the functions of the device, its limitations and its procedures for use are well understood by users to ensure correct use and to reduce risks of the adverse effects of continuous FHRM.

## Additional files

Additional file 1: Interview guide focus group discussions. (PDF 84 kb)

Additional file 2: Interview guide semi-structured interviews. (PDF 292 kb)

## Abbreviations

CS: Cesarean-section
CTG: Cardiotocography
FGD: Focus group discussion
FHR: Fetal heart rate
FHRM: Fetal heart rate monitoring
FIGO: International federation of obstetrics and gynaecology
IA: Intermittent auscultation
IDI: in-depth interview
