# Supplementary material for: Perceptions and experiences of skilled birth attendants on using a newly developed strap-on electronic fetal heart rate monitor in Tanzania
Source: BMC Pregnancy Childbirth. 2019 May 10;19:165. doi: 10.1186/s12884-019-2286-7 (PMC6511185; doi:10.1186/s12884-019-2286-7)
Supplement: Supplementary file 1 — Interview guide focus group discussions. (PDF 84 kb) [file 12884_2019_2286_MOESM1_ESM.pdf]

FOCUS GROUP DISCUSSION
Fetal heart rate monitoring

Objectives:
- Capture health care workers attitudes about labor monitoring and the different methods used.

Introduction to the session: *This session is to discuss labor monitoring and the different methods used. Please note that there are no right and wrong answers. We will be asking you nine questions. Before we start, does anyone has any objections to the session being recorded?*

1. In your opinion, is it important to measure fetal heart rate? (why?)
2. When is it important to measure fetal heart rate?
3. What are the other ways to monitor a labor except from measuring the fetal heart rate?
4. Which method would you say you use the most frequently to monitor a labor?
5. What do you trust the most to determine FHR- pinnard or Moyo/doppler? why?
6. What are some of the positive and negative aspects of using the partograph?
7. How do you define fetal distress?
8. There is a function on Moyo measuring maternal fetal heart rate. Could you tell me when you last used the function and what was the reason you wanted to know the mother´s pulse.
9. What are the comments you have heard from the mothers about Moyo?

To close the session:
As we are coming to the end of this session, is there something that you would you like to say that you did not get a chance to say during the session or do you have any questions? (pause) *We thank you for taking the time to participate, and please do not hesitate to contact us.*
