# Supplementary material for: Perceptions and experiences of skilled birth attendants on using a newly developed strap-on electronic fetal heart rate monitor in Tanzania
Source: BMC Pregnancy Childbirth. 2019 May 10;19:165. doi: 10.1186/s12884-019-2286-7 (PMC6511185; doi:10.1186/s12884-019-2286-7)
Supplement: Supplementary file 2 — Interview guide semi-structured interviews. (PDF 292 kb) [file 12884_2019_2286_MOESM2_ESM.pdf]

In-depth interview guide
Skilled birth attendants Tanzania
February 2017

Objectives:
- Capture participants experiences using Moyo
- Capture how the participants feel that using the device affects patient care.
- Capture suggestions for improvements/changes to the device.

Introduction to the session: *This session is to discuss your experience using Moyo. Please note that there are no right and wrong answers. We will be asking you ten questions. Before we start, do you have any objections to the session being recorded?*

1. Can you tell me about a regular day at work?
2. Moyo has been introduced at this facility, what would you say are the reasons it has been introduced?
3. in your work what do you consider the indications to put Moyo on, for how long do you leave it on, what are the indications to take it off?
4. Would you say that any your actions to alerts from Moyo of abnormal fetal heart rate are different from when you detect an abnormal FHR using other monitoring techniques such as pinnard or doppler?
5. In what way do you feel using Moyo affect your work load and stress level?
6. Compared to using other methods of fetal heart rate monitoring- do you feel using Moyo changes the way you interact with your patients?
7. If it is anything you would like to change about Moyo, what would it be?
8. What are the challenges associated with the use of Moyo?
9. There is a function on Moyo measuring maternal fetal heart rate. Could you tell me when you last used the function and what was the reason you wanted to know the mother´s pulse.
10. What are the comments you have heard from the mothers about Moyo?

To close the session:
As we are coming to the end of this session, is there something that you would you like to say that you did not get a chance to say during the session? (pause) *We thank you for taking the time to participate, and please do not hesitate to contact us.*
